# Intraocular pressure and its normal range adjusted for ocular and systemic parameters. The Beijing Eye Study 2011

**DOI:** 10.1371/journal.pone.0196926

**Published:** 2018-05-17

**Authors:** Ya Xing Wang, Liang Xu, Wen Bin Wei, Jost B. Jonas

**Affiliations:** 1 Beijing Institute of Ophthalmology, Beijing Tongren Eye Center, Beijing Tongren Hospital, Capital Medical University, Beijing Ophthalmology and Visual Science Key Lab, Beijing, China; 2 Depart of Ophthalmology, Beijing Tongren Eye Center, Beijing Tongren Hospital, Capital Medical University, Beijing Ophthalmology and Visual Science Key Lab, Beijing, China; 3 Department of Ophthalmology, Medical Faculty Mannheim of the Ruprecht-Karls-University of Heidelberg, Mannheim, Germany; Oregon Health and Science University, UNITED STATES

## Abstract

**Purpose:**

To examine the distribution of intraocular pressure (IOP) in a normal population and the associations of IOP with other ocular and systemic parameters.

**Methods:**

Out of 3468 participants of the population-based cross-sectional Beijing Eye Study 2011 we selected those individuals without glaucomatous optic neuropathy. The study particpants underwent a detailed ophthalmologic and systemic examination. IOP was measured by air puff non-contact tonometry.

**Results:**

The study included 3135 eyes of 3135 participants with a mean age of 64.1 ± 9.6 years (mean ± standard deviation). The mean IOP was 14.7 ± 2.8 mmHg. The 95% percentile and 97.5% percentile of the IOP distribution decreased from 20 mmHg / 21 mmHg in individuals aged 40 to 54 years to 18 mmHg / 19 mmHg in individuals aged ≥80 years. In multivariable analysis, higher IOP was associated with the systemic parameters of younger age (*P*<0.001), higher blood concentration of glucose (*P* = 0.03) and triglycerides (*P*<0.001), higher diastolic blood pressure (*P*<0.001), higher pulse rate (*P* = 0.003) and higher quantity of alcohol consumption (*P* = 0.004), and with the ocular parameters of larger central corneal thickness (*P*<0.001), more myopic refractive error (*P* = 0.01) and steeper anterior corneal curvature radius (*P* = 0.006). IOP decreased significantly by 0.50 mmHg and 0.76 mmHg for each increase in age by 10 years and each increase in corneal curvature radius by 1.0 mm, respectively. The range of the mean ± 2 standard deviations of the IOP adjusted for the parameters of the multivariable model was 9.0 to 18.1 mmHg versus 9.2–20.2 mmHg for the unadjusted IOP. In the age group of 50 to 55 years, the age-adjusted IOP range (mean ± 2 standard deviations) was 9 to 18 mmHg, and in the age group of ≥75 years, it was 8 to 18 mmHg.

**Conclusions:**

IOP physiologically depends on a multitude of systemic and ocular factors including age and blood pressure. These physiological associations of the IOP may be taken into account in the definition of the normal range of the IOP.

## Introduction

Glaucoma can be defined as an optic neuropathy in eyes in which the intraocular pressure (IOP) is higher than the pressure tolerance of the retinal ganglion cells axons in the lamina cribrosa in the optic nerve head [1]. The traditional criterion for the presence of glaucoma was an IOP higher than 21 mm Hg [2]. Subsequent studies revealed however, that the IOP was within the previously defined normal range of 10 mm Hg to 21 mm Hg in many glaucoma patients although these patients showed morphological and functional features of optic nerve damage such as increased optic disc cupping and loss of neuroretinal rim, development and enlargement of parapapillary beta zone, and wedge-shaped visual field defects including nasal steps and Bjerrum scotomas [3, 4]. The combination of glaucomatous optic nerve damage and statistically normal IOP was called normal-pressure glaucoma. It was found to be present particularly in East Asian patients [5, 6]. Consequently, the Japanese population-based Tajimi study revealed that the IOP was 21 mmHg or less in 92% of Japanese patients with primary open-angle glaucoma [3].

The definition of the normal range of IOP was based on a large-scaled hospital-based study performed by Leydhecker and colleagues in 1958, and on an investigation conducted by Hollows and Graham in 1966, who used a rigorous population-based sampling method [2, 7]. These studies however had limitations since the study populations included only Caucasians and since associations of the IOP with other ocular and systemic factors in the normal population were not taken into account. Investigations performed later revealed that in the normal population higher IOP was associated with systemic parameters such higher diastolic blood pressure, higher pulse rate, higher body mass index and a higher level of education, and with ocular factors such as a higher corneal refractive power, a lower central corneal thickness and a longer axial length [8–18]. In some studies focusing on Asian populations, the IOP was also found to be associated with younger age [3–17].

If a variable such as the IOP is taken to define the presence of a disease and if this variable in the normal population shows physiological associations with a variety of other parameters, primarily not correlated with the disease itself, these associations should be taken into account when the normal range of the variable is defined and used for the definition of a disease. Previous studies on normal-pressure glaucoma did not always consider the physiologic associations between IOP and the other ocular and systemic parameters [3, 5, 19]. The notion is therefore that the normal range of IOP may be re-defined by taking into account the associations between IOP and other ocular and systemic parameters before the IOP readings are used as one among several parameters to diagnose glaucoma. We therefore conducted this study to re-assess correlations between IOP readings and other variables and to re-define the normal range of IOP in a non-glaucomatous population recruited in population-based cohort. We excluded patients with glaucoma in our study since the investigation was focused on the physiological associations of the IOP which if taken into account could potentially allow an improved separation of normal eyes from glaucomatous eyes based on the IOP readings.

## Methods

The Beijing Eye Study 2011 is a population-based cross-sectional study which was carried out in a central region of Beijing (Haidan region) and in a rural area South of Beijing (village area of Yufa, Daxing District). The Medical Ethics Committee of the Beijing Tongren Hospital approved the study and written informed consent was obtained from all participants. All inhabitants of the study regions with an age of ≥ 50 years (n = 4403 individuals) were eligible to participate.

The study population included 3468 individuals (1963 (56.6%) women) (response rate: 78.8%) with a mean age of 64.6 ± 9.8 years (median, 64 years; range, 50–93 years). The series of examination started with an interview containing standardized questions on the socioeconomic background including the family status, level of education (as differentiated between illiteracy, half illiteracy, primary school, high school, and college or higher level) and self-reported income, on previous and current consumption of alcohol, smoking habits, and on the history of major systemic diseases such as arterial hypertension, diabetes mellitus, hyperlipidemia, cardiovascular disorders and cerebral infarction. We measured the body height and weight, the blood pressure and the concentrations of lipids, glucose, glycosylated hemoglobin HbA1c, creatinine and C-reactive protein in blood samples obtained under fasting conditions. Diabetes mellitus was defined as a plasma glucose concentration of ≥7.0 mmol/L or by a self-reported history of physician-based diagnosis of diabetes mellitus or by a history of medical treatment of diabetes. Arterial hypertension was characterized by a systolic blood pressure of ≥140 mm Hg and/or a diastolic blood pressure of ≥90 mm Hg, and/or a self-reported current treatment of arterial hypertension. The mean blood pressure was calculated as the sum of the diastolic blood pressure plus one third of the pressure amplitude.

The ophthalmological examinations consisted of refractometry, non-contact tonometry (CT-60 computerized tonometer, Topcon Ltd., Japan), slit lamp examination of the anterior and posterior segment of the eye, digital photography of the cornea, lens, macula and optic disc (fundus camera Type CR6-45NM; Canon Inc, Tokyo, Japan), spectral domain optical coherence tomography (Spectralis®, Heidelberg Engineering Co., Heidelberg, Germany) of the macula and optic nerve head, and biometry for measurement of the central corneal thickness and corneal curvature, anterior chamber depth, lens thickness and total axial length (Lenstar 900® Optical Biometer, Haag-Streit, 3098 Koeniz, Switzerland). Tonometry and refractometry carried out by an experienced technician were performed three times, and the means of the measurements were taken for further statistical analysis.

The degree of cataract was determined using the lens photographs. The degree of nuclear opacities was assessed in 6 grades using the grading system of the Age-Related Eye Disease Study [20]. On the retro-illuminated lens photographs we determined the degree of cortical cataract and posterior subcapsular cataract [21]. Examining the fundus photographs, we differentiated retinal vein occlusions into central retinal vein occlusions and branch retinal vein occlusions. Central retinal vein occlusions were characterized by retinal edema, optic disc hyperemia or edema, scattered superficial or deep retinal hemorrhages and venous dilation in their acute stage, and by occluded and sheathed retinal veins or vascular anastomoses at the optic disc in their chronic stage. Branch retinal vein occlusions showed a localized retinal edema, superficial and deep retinal hemorrhages, intraretinal microvascular abnormalities or anastomotic vessels, and venous dilatation or venous sheathing within a sector of the retina corresponding to the obstructed vein [22]. Diabetic retinopathy was defined according to the Early Treatment of Diabetic Retinopathy Study (ETDRS) criteria [23]. The minimum criterion for diagnosis of diabetic retinopathy was the presence of at least one microaneurysm. Cerebrospinal fluid pressure (CSFP) was estimated using the formula: “CSFP [mmHg] = 0.44 x Body Mass Index [kg/m^2^] + 0.16 x Diastolic Blood Pressure [mmHg]– 0.18 x Age [Years] - 1.91” [24]. Glaucomatous optic neuropathy was defined by absolute criteria each of which were sufficient for the diagnosis of glaucoma and by relative criteria. The absolute criteria included a notch in the neuroretinal rim in the temporal inferior region and/or the temporal superior region, so that the inferior-superior-nasal-temporal (ISNT)-rule of the neuroretinal rim shape was not fulfilled (in eyes with an optic cup sufficiently large to allow an assessment of the neuroretinal shape), localized retinal nerve layer defects which could not be explained by any other cause than glaucoma, and an abnormally large cup in relation to the size of the optic disc [25, 26]. Relative criteria for the diagnosis of glaucomatous optic neuropathy were a neuroretinal rim which was markedly thinner in the inferior disc region compared with the superior disc region, even if the smallest part of the neuroretinal rim was located in the temporal horizontal disc region; a diffuse decrease in the visibility of the retinal nerve fiber layer (particularly in eyes with small optic discs), if the background pigmentation of the eye allowed an assessment of the retinal nerve fiber layer and if there was no other reason than glaucoma for the retinal nerve fiber layer loss; a marked diffuse thinning and/or focal thinning of the retinal arteries if there was no other reason than glaucoma for retinal vessel thinning; and an optic disc hemorrhage, if there was no other reason for a disc bleeding such as retinal vessel occlusions. If none of the absolute glaucoma criteria were fulfilled, the diagnosis of glaucoma required that at least two relative criteria had to be fulfilled, among them had to be a suspicious neuroretinal rim shape in eyes with an optic cup large enough for the assessment of the rim shape; or at least two relative criteria had to be positive including the occurrence of an optic cup in a small optic disc which usually would not show cupping. Using digital fundus photographs, the assessment of glaucomatous optic neuropathy was carried out by two senior graders (YXW, JBJ). In case of disagreement, the optic disc photographs were re-assessed up to three times, until eventually both graders agreed upon the diagnosis.

The only exclusion criterion for the present study was the presence of glaucoma. The data of the right eye of each study participant was taken for statistical analysis.

### Statistical analysis

The statistical analysis was carried out using a software package (SPSS for Windows, version 22.0, IBM-SPSS, Chicago, IL). In a first step, we calculated the mean values ± standard deviations of the main parameters, and we determined the normal range of the IOP defined as the mean ± 2 standard deviations. In a second step, we assessed associations between the IOP and other ocular and systemic parameters in a univariate mode. In a third step, we re-tested in a multivariate analysis the associations between IOP and all parameters which were significantly associated with the IOP in the univariate analysis. Out of the list of independent parameters, we first dropped step by step parameters with a high collinearity (defined as a variance inflation factor (VIF) of higher than 5), before we deleted those parameters which were no longer significantly correlated with the IOP. In a fourth step, we used the final model to adjust the raw IOP readings for their dependency on the parameters of the final model. We applied the general formula “IOP_Adjusted_ = IOP_Measured_−((Non-Standardized Correlation Coefficient B) x (Parameter–Mean Value of the Parameter in the Study Population)) + Intercept of the Regression Line of the Y-Axis”. We determined the standardized regression coefficient beta, the non-standardized regression coefficient B, and the 95% confidence intervals (CI). All *P-*values were two-sided and considered statistically significant when the values were less than 0.05.

## Results

Out of the whole population of the Beijing Eye Study (n = 3468), 3135 eyes of 3135 participants (90.4%) fulfilled the inclusion criteria. The age (mean ± standard deviation) was 64.1 ± 9.6 years (range: 50–93 years), and the mean refractive error was -0.17 ± 2.03 diopters (range: -21.0 to +7.00 diopters) (Table 1). Axial length ranged between 18.96 mm and 30.88 mm (mean: 23.2 ± 1.1 mm). The group of individuals included into the study as compared with the group of persons excluded was significantly younger (64.1 ± 9.6 years versus 71.7 ± 9.2 years; *P* <0.001), less myopic (-0.17 ± 2.03 diopters versus -1.00 ± 3.11 diopters; *P* = 0.002), and had a shorter axial length (23.2 ± 1.1 mm versus 23.7 ± 1.4 mm; *P* = 0.02).

**Table 1:** Baseline characteristics of the individuals included into the present investigation and of those participants of the Beijing Eye Study who were not included into the present investigation.

Parameters	Individuals Included	Individuals excluded	*P*-Value
Age (Years)	64.0 ± 9.6	71.2 ± 9.2	<0.001
Gender (Male%)	43.3%	50.0%	0.060
Rural / Urban Habitation (Rural %)	46.9%	26.9%	<0.001
Level of Education	3.9 ± 1.0	4.0 ± 1.2	0.318
Axial Length (mm)	23.2 ± 1.1	23.7 ± 1.4	<0.001
Central corneal thickness (μm)	532 ± 32	536 ± 34	0.166
Refractive Error (Diopters)	-0.18 ± 2.03	-1.03 ± 3.11	<0.001
Body Mass Index (kg/m^2^)	25.6 ± 3.8	24.5 ± 3.8	<0.001
Systemic blood pressure (mmHg)	130 ± 20	131 ± 22	0.429
Diastlic blood pressure (mmHg)	70 ± 12	67 ± 11	0.001
Pulse Rate (Beats/min)	73 ± 10	74 ± 11	0.075

Presented as mean ± standard deviation or percetage

**Fig 1:**
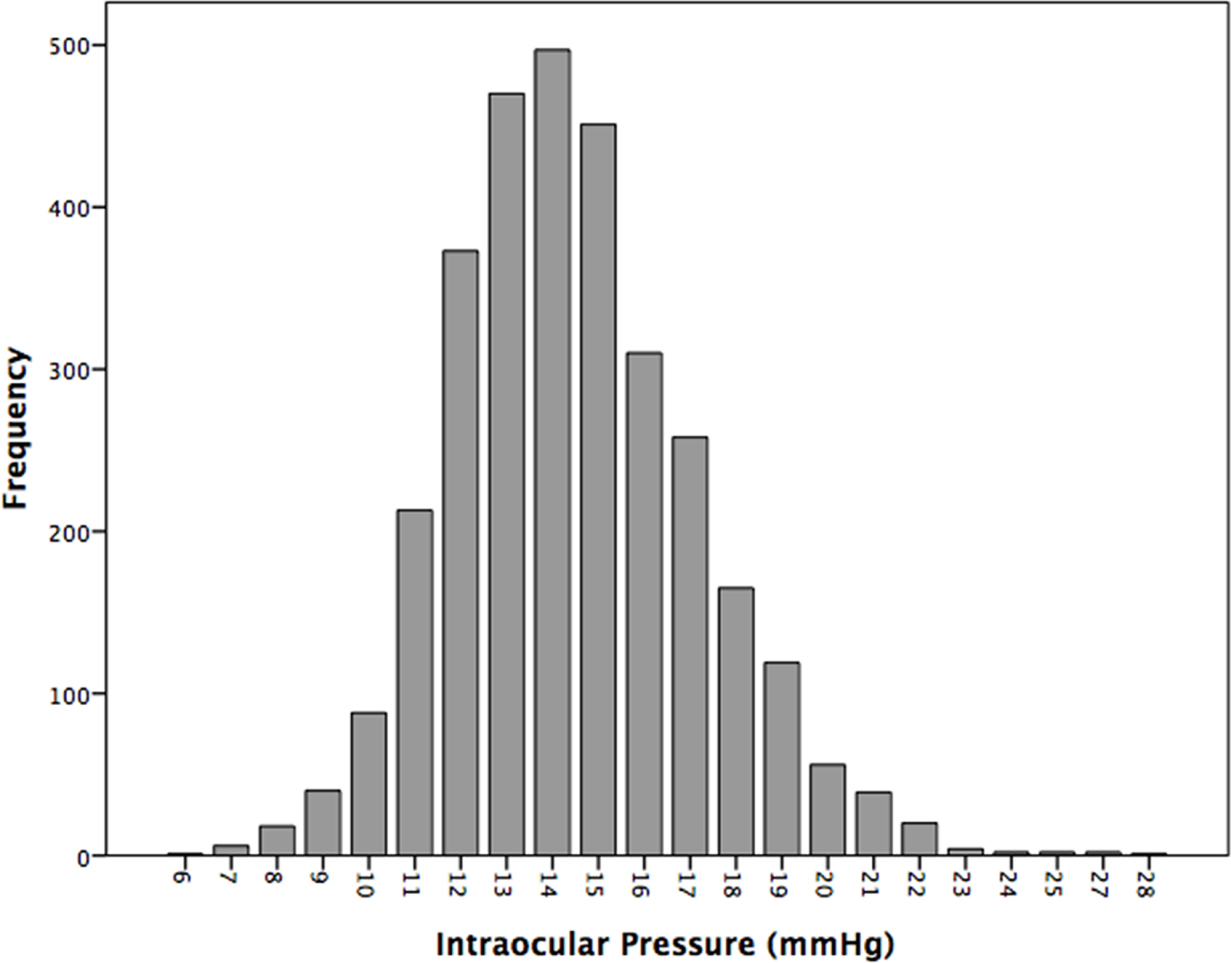
Histogram showing the distribution of intraocular pressure (raw data) in non-glaucomatous participants of the Beijing Eye Study 2011.

**Fig 2:**
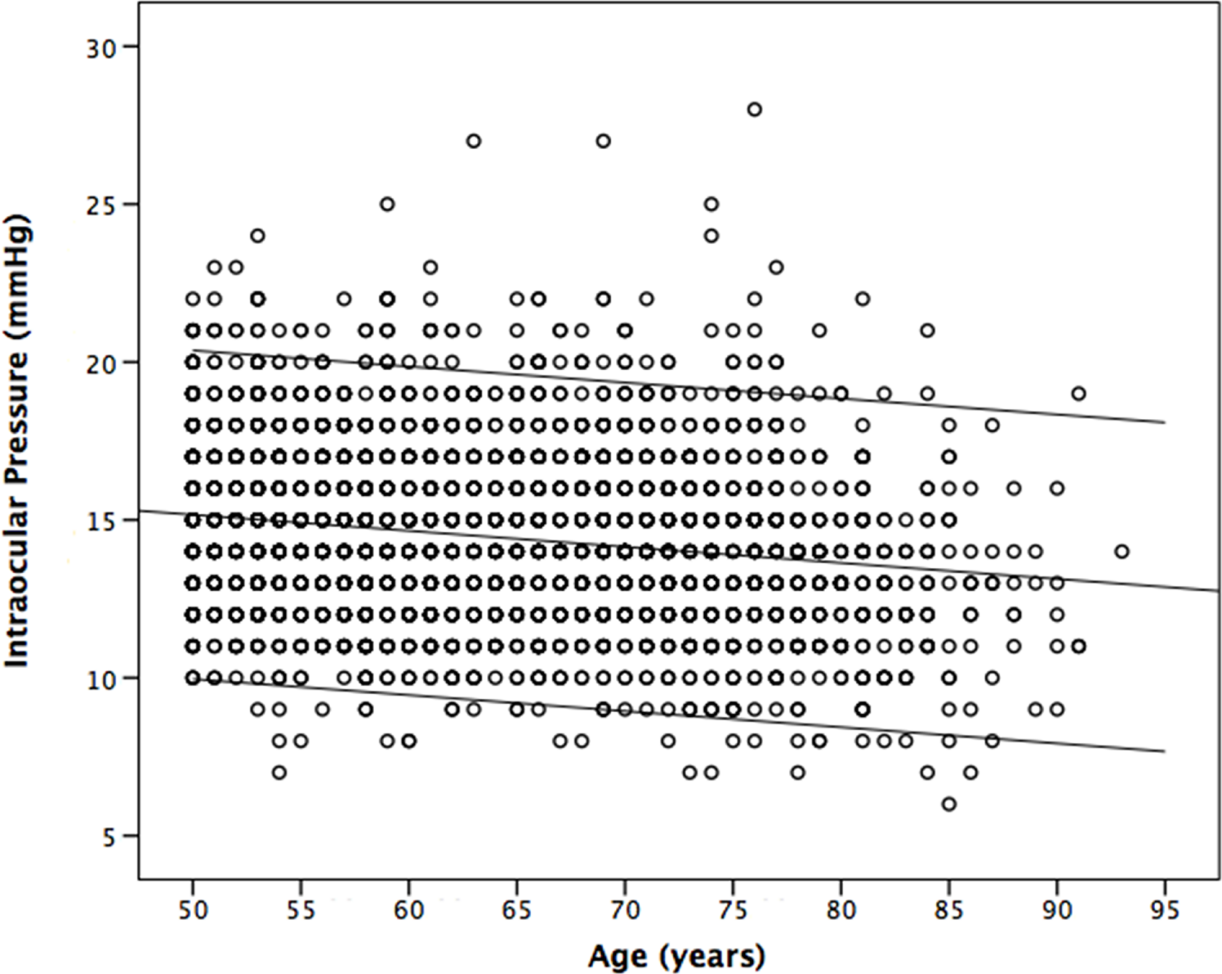
Scattergram showing the distribution of IOP versus age in non-glaucomatous participants of the Beijing Eye Study 2011.

**Fig 3:**
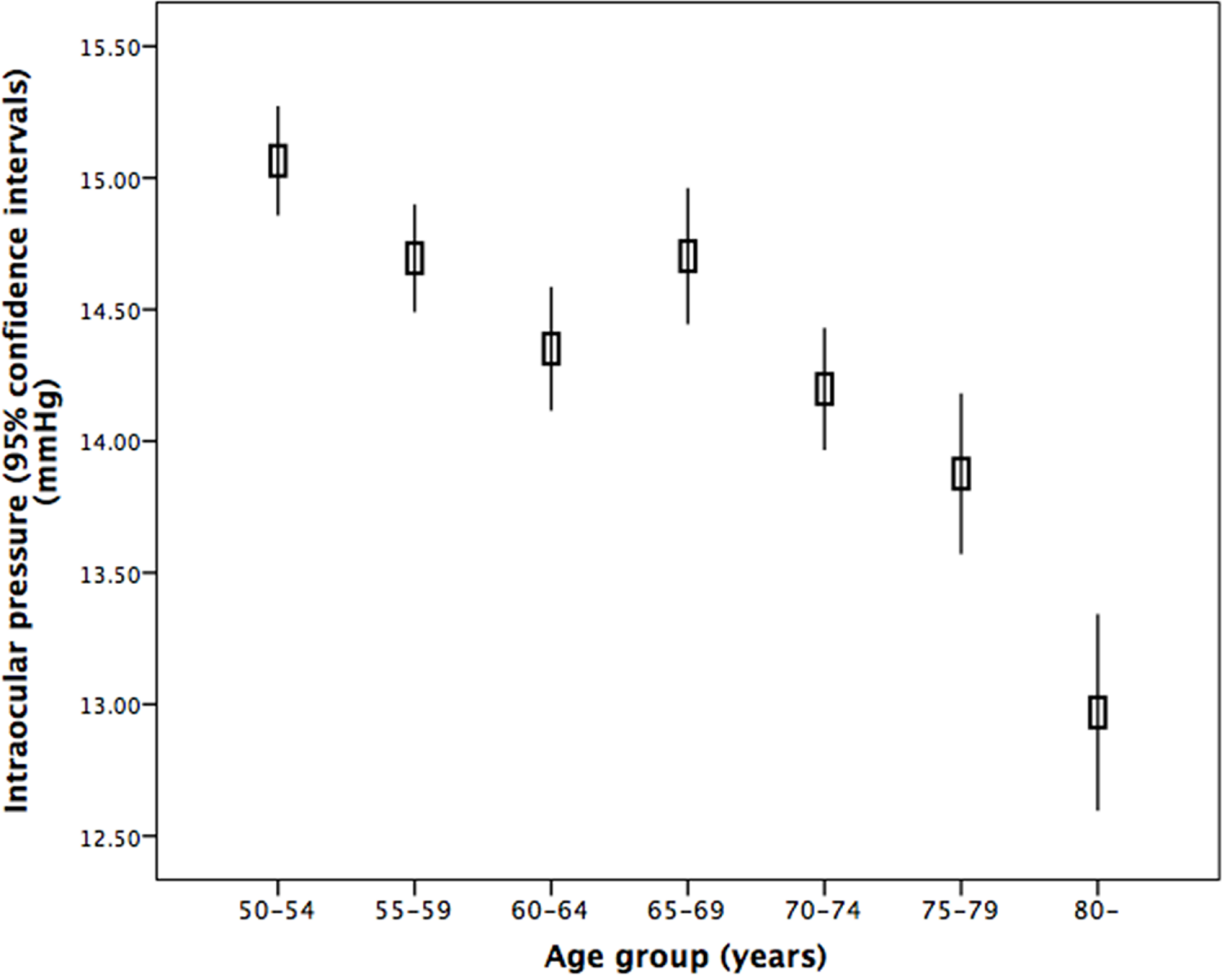
Graph showing the distribution of intraocular pressure stratified by age in non-glaucomatous participants of the Beijing Eye Study 2011.

In the whole study population, the mean IOP was 14.45 ± 2.70 mmHg (mean ± standard deviation). The IOP range defined as the mean ± 2 standard deviations was 9.17–19.74 mmHg) (Fig 1). The IOP decreased significantly with older age (Fig 2). Stratified by age groups, the mean ± standard deviation and the range of the mean ± 2 standard deviations of the IOP decreased from 15.2 ± 2.7 mmHg (range: 11.2–20.6 mmHg) to 13.3 ± 2.9 mmHg (range: 7.6–19.0 mmHg) (Table 2) (Fig 3). The 95% percentile and 97.5% percentile of the IOP was 20 mmHg / 21 mmHg, 19 mmHg/ 20 mmHg, 19 mmHg/ 20 mmHg, 20 mmHg/ 20 mmHg, 19 mmHg/ 20 mmHg, 19 mmHg/ 20 mmHg, 18 mmHg/ 19 mmHg, for the age groups of 50 to 54 years, 55 to 59 years, 60 to 64 years, 65 to 69 years, 70 to 74 years, 75 to 79 years, and ≥80 years, respectively.

**Table 2:** Intraocular pressure in the non-glaucomatous participants of the Beijing Eye Study 2011, stratified by age.

		Intraocular pressure (mmHg)				
Age group (year)	n.	Mean	Standard deviation	95% Confidence interval	95% Percentile	97.5% Percentile
50 to <55	631	15.1	2.7	9.9–20.3	20	21
55 to <60	606	14.7	2.6	9.7–19.7	19	20
60 to <65	476	14.4	2.6	9.2–19.5	19	20
65 to <70	444	14.7	2.8	9.3–20.1	20	20
70 to <75	460	14.2	2.5	9.2–19.2	19	20
75 to <80	323	13.9	2.8	8.4–19.3	19	20
80+	195	13.0	2.6	7.8–18.2	18	19

**Fig 4:**
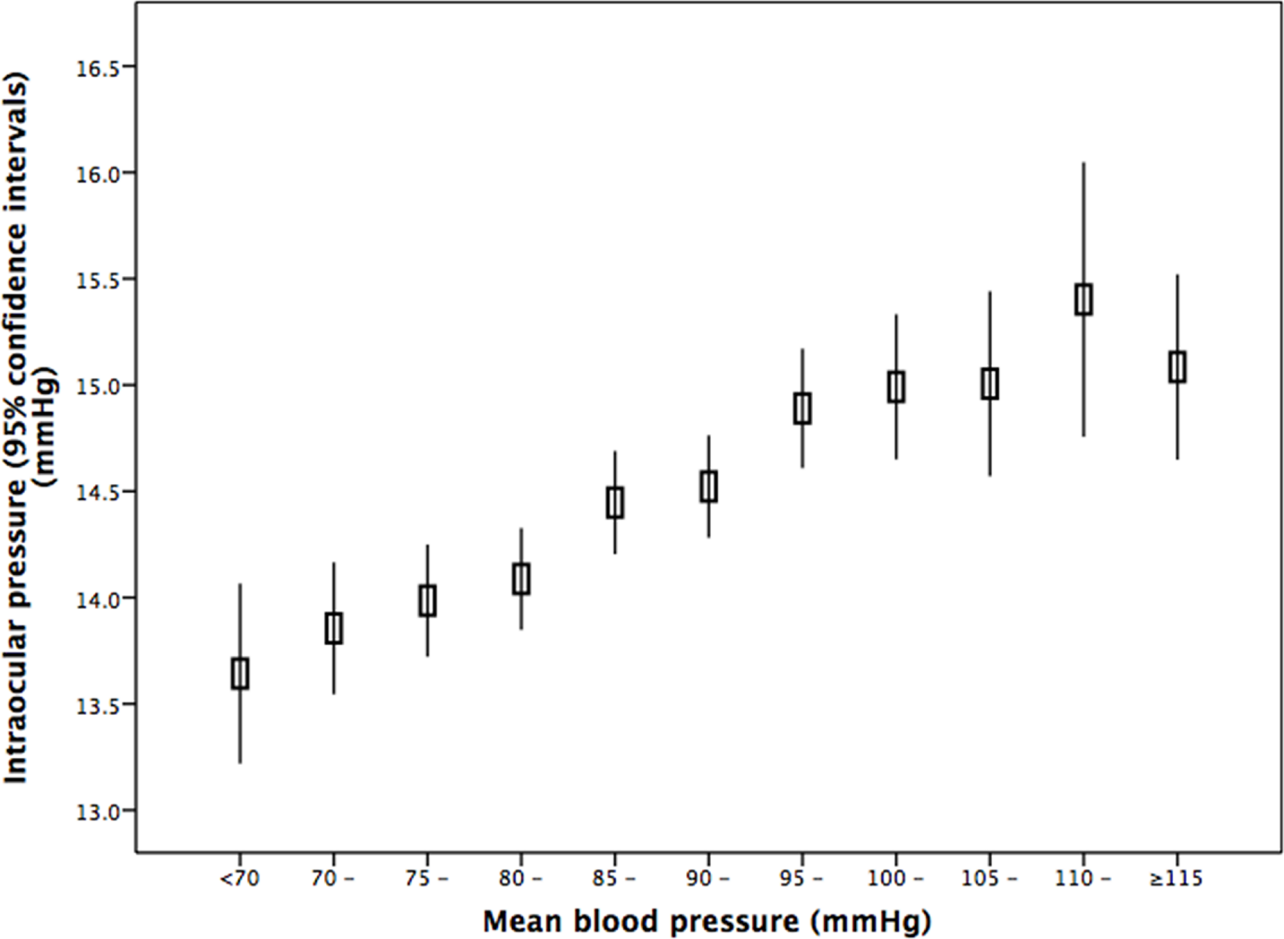
Graph showing the distribution of intraocular pressure stratified by mean blood pressure in non-glaucomatous participants of the Beijing Eye Study 2011.

**Fig 5:**
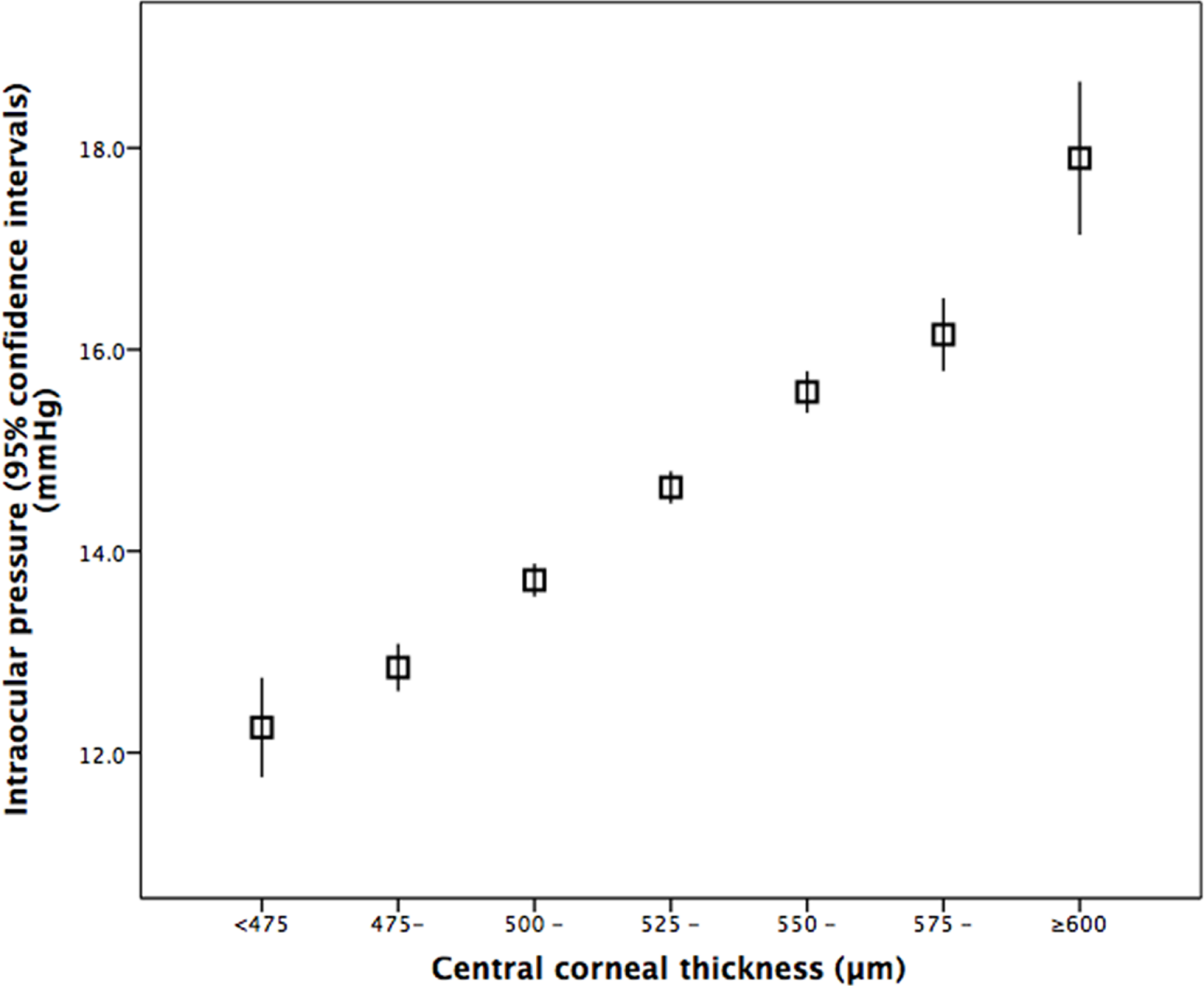
Graph showing the distribution of intraocular pressure stratified by central corneal thickness in non-glaucomatous participants of the Beijing Eye Study 2011.

In univariate analysis, higher IOP was associated with the systemic variables of younger age (*P*<0.001) (Table 2) (Figs 2 and 3), rural region of habitation (*P*<0.001), heavier body weight (*P*<0.001), higher body mass index (*P*<0.001), larger waist and hip circumference (*P*<0.001), higher blood concentrations of glucose (*P*<0.001), glycosylated hemoglobin HbA1c (*P*<0.001), low-density lipoproteins (*P*<0.001), triglycerides (*P*<0.001) and cholesterol (*P*<0.001), lower blood concentrations of high-density lioproteins (*P*<0.001), higher prevalence of diabetes mellitus (*P*<0.001), higher systolic, diastolic and mean blood pressure (*P*<0.001) (Fig 4), higher prevalence of arterial hypertension (*P*<0.001), higher pulse rate (*P*<0.001), higher number of smoking package years (*P*<0.001), smoking ever (*P*<0.001), and higher frequency and quantity of alcohol consumption (*P*<0.001). Higher IOP was correlated with the ocular parameters of thicker cornea (*P*<0.001) (Fig 5), more myopic refractive error (*P* = 0.022), shallower anterior chamber depth (*P* = 0.002), thinner lens thickness (*P* = 0.001), and higher prevalence of diabetic retinopathy (*P* = 0.042) (Table 3).

**Table 3:** Associations (univariate analysis) of intraocular pressure with systemic parameters and ocular variables in the non-glaucomatous participants of the Beijing Eye Study 2011.

Parameter	*P*-Value	B	95% CI of B	Beta
***Systematic Parameters*:**				
Age (Years)	<0.001	-0.05*	-0.06, -0.04	-0.18
Gender (Men/Women)	0.128	-0.15	-0.34, 0.04	-0.03
Rural / Urban Region of Habitation	<0.001	-0.49*	-0.68, -0.30	-0.09
Level of Education	0.53	-0.03	-0.12, 0.06	-0.11
Body Height (cm)	0.055	0.01	0, 0.02	0.03
Body Weight (kg)	<0.001	0.02*	0.01, 0.03	0.09
Body Mass Index (kg/m^2^)	<0.001	0.06*	0.03, 0.08	0.08
Waist Circumference (cm)	<0.001	0.02*	0.01, 0.03	0.08
Hip Circumference (cm)	<0.001	0.02*	0.01, 0.04	0.06
Blood Concentration Glucose (mmol/L)	<0.001	0.18*	0.10, 0.25	0.10
Glycosylated Hemoglobin HbA1c (%)	<0.001	0.22*	0.10, 0.34	0.08
Diabetes Mellitus	<0.001	0.47*	0.21, 0.74	0.06
Blood Concentration High-Density Lipoprotein (mmol/L)	0.008	-0.38	-0.67, -0.10	-0.06
Blood Concentration Low-Density Lipoprotein (mmol/L)	<0.001	0.16*	0.03, 0.28	0.05
Blood Concentration Triglycerides (mmol/L)	<0.001	0.26*	0.17, 0.35	0.11
Blood Concentration Cholesterol (mmol/L)	<0.001	0.16*	0.05, 0.28	0.06
Systolic Blood Pressure (mm Hg)	<0.001	0.02*	0.01, 0.02	0.12
Diastolic Blood Pressure (mm Hg)	<0.001	0.04*	0.03, 0.05	0.19
Mean Blood Pressure (mm Hg)	<0.001	0.03*	0.03, 0.04	0.17
Arterial Hypertension	<0.001	0.31*	0.12, 0.50	0.06
Pulse Rate (Beats/min)	<0.001	0.03*	0.02, 0.03	0.09
Smoking Package Years	<0.001	0.01*	0.01, 0.02	0.08
Smoking (Ever)	<0.001	0.45*	0.24, 0.65	0.08
Alcohol Consumption Frequency	<0.001	0.11*	0.15, 0.30	0.16
Alcohol Consumption Quantity	<0.001	0.23*	0.16, 0.31	0.11
Aspirin Intake	0.147	-0.16	-0.37, 0.06	-0.03
***Ocular Parameters*:**				
Refractive Error (Diopters)	0.022	-0.05	-0.10, -0.01	-0.04
Central Corneal Thickness (μm)	<0.001	0.04*	0.03, 0.04	0.44
Anterior Corneal Curvature (mm)	0.19	-0.26	-0.64, 1.27	-0.02
Anterior Chamber Depth (mm)	0.002	-0.31	-0.51, -0.11	-0.06
Lens Thickness (mm)	0.001	-0.48*	-0.77, -0.18	-0.06
Axial Length (mm)	0.54	0.03	-0.06, 0.12	0.11
Optic Disc Area (mm^2^)	0.838	-0.04	-0.38, 0.31	-0.06
Retinal Nerve Fiber Layer Thickness (μm)	0.07	-0.01	-0.02, 0	-0.03
Early Age-Related Macular Degeneration	0.086	-0.30	-0.65, -0.04	-0.03
Diabetic Retinopathy	0.042	0.57	0.02, 1.12	0.04
Retinal Vein Occlusion	0.46	-0.22	-0.81, 0.37	0.01

95% CI: 95% Confidence Interval; B: Regression Coefficient; Beta: Standardized Correlation Coefficient

* Correlation is significant after Bonferroni correction for conducting multiple comparisons (P<0.0014)

Education was graded as 0: illiteracy; 1: half illiteracy; 2: primary school; 3: high school; 4: college or above college level.

Alcohol Consumption Frequency was graded as, 0: never drank alcohol; 1: former alcohol consumption but quitted; 2: alcohol consumption less than once per month; 3: alcohol consumption once per month; 4: alcohol consumption 2–3 times per month; 5: alcohol consumption once per week; 6: alcohol consumption 2–3 times per week; 7: alcohol consumption every day

The multivariate analysis included IOP as the dependent variable and all those parameters as independent variables which were significantly associated with the IOP in the univariate analysis. Due to collinearity, we first dropped the parameters with a VIF higher than 5, including body weight (VIF: 141), body mass index (VIF: 107), blood concentration of low-density lipoproteins (VIF: 28.7), blood concentration of cholesterol (VIF: 35.4), blood concentration of high-density lipoproteins (VIF: 6.5), and waist circumference (VIF: 5.1). Due to lack of statistical significance we then dropped step by step the parameters of rural/urban habitation (*P* = 0.86), lens thickness (*P* = 0.87), anterior chamber depth (*P* = 0.46), ever smoking (*P* = 1.00), glycosylated hemoglobin HbA1c (*P* = 0.59), blood concentration of glucose (*P* = 0.85), hip circumference (*P* = 0.16), alcohol consumption frequency (*P* = 0.82), history of arterial hypertension (*P* = 0.12), and smoking package years (*P* = 0.20). In the final model, higher IOP was associated with the systemic parameters of younger age (*P*<0.001), higher blood concentration of triglycerides (*P*<0.001), higher mean blood pressure (*P*<0.001), higher pulse rate (*P* = 0.001), higher drinking quantity of alcohol (*P* = 0.006) and higher prevalence of diabetic mellitus (*P* = 0.004), and with the ocular parameters of larger central corneal thickness (*P*<0.001), more myopic refractive error (*P* = 0.01) and steeper anterior corneal curvature radius (*P*<0.001) (Table 4). If refractive error was replaced by axial length, higher IOP was significantly associated with longer axial length (*P* = 0.016; beta: 0.05; B: 0.14; 95%CI: 0.03, 0.25).

**Table 4:** Associations (multivariable analysis) of intraocular pressure with systemic parameters and ocular variables in the non-glaucomatous participants of the Beijing Eye Study 2011 (n = 3135).

Parameter	*P*-Value	B	95% CI of B	Beta	VIF
Age (Years)	<0.001	-0.05	-0.06, -0.04	-0.17	1.04
Mean Blood Pressure (mm Hg)	<0.001	0.03	0.02, 0.04	0.15	1.03
Pulse Rate	0.001	0.02	0.01, 0.03	0.06	1.03
Blood Concentration of Triglycerides (μmol/L)	<0.001	0.17	0.08, 0.25	0.07	1.03
Diabetes or not (based on history plus fast glucose)	0.004	0.41	0.13, 0.68	0.06	1.05
Alcohol Consumption Quantity	0.006	0.11	0.03, 0.18	0.05	1.05
Refractive Error (Diopters)	0.010	-0.07	-0.12, -0.02	-0.05	1.00
Central Corneal Thickness (μm)	<0.001	0.04	0.035, 0.041	0.46	1.03
Anterior Corneal Curvature Radius (mm)	<0.001	-0.73	-1.14, -0.33	-0.07	1.04

95% CI: 95% Confidence Interval; B: Unstandardized Coefficient; Beta: Standardized Correlation Coefficient; VIF: Variance Inflation Factor

**Fig 6:**
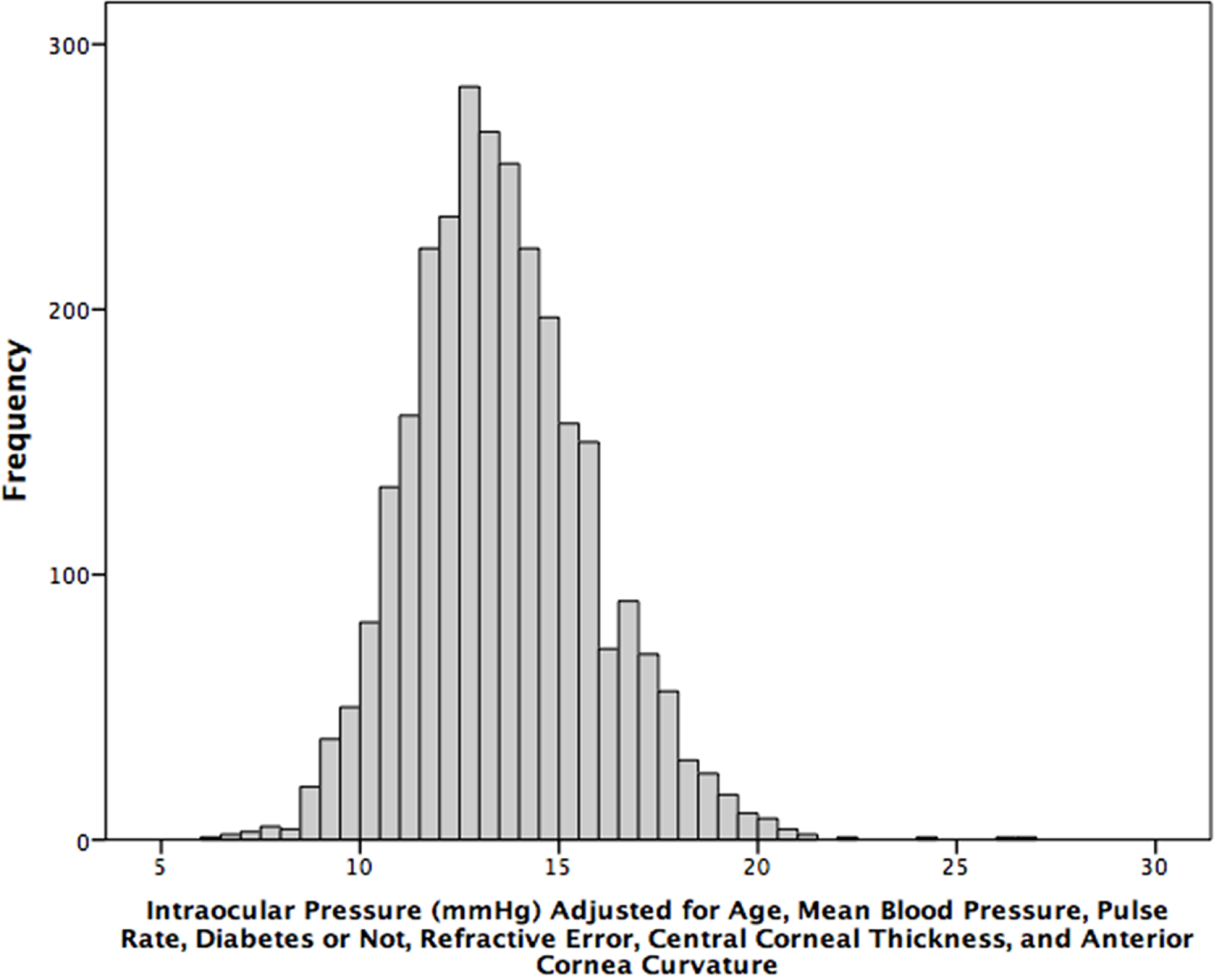
Histogram showing the distribution of intraocular pressure adjusted age, body height, diastolic blood pressure, pulse rate, refractive error, central corneal thickness and anterior corneal curvature radius in non-glaucomatous participants of the Beijing Eye Study 2011.

If the list of independent variables in the multivariate analysis included only parameters which showed the highest standardized correlation coefficients and which could be assessed under routine clinical conditions, i.e., age, diastolic blood pressure, pulse rate, history of diabetes, refractive error, central corneal thickness and anterior corneal curvature radius, higher IOP was correlated (correlation coefficient r: 0.53) with younger age (*P*<0.001), higher mean blood pressure (*P*<0.001), higher pulse rate (*P*<0.001), higher prevalence of diabetic mellitus (*P* = 0.004), more myopic refractive error (*P* = 0.001), larger central corneal thickness (*P*<0.001) and flatter cornea (*P*<0.001) (Table 5). If refractive error was replaced by axial length, higher IOP was significantly associated also with longer axial length (*P* = 0.016; beta: 0.05; B: 0.14; 95%CI: 0.03, 0.25). Using the model, the equation for adjusting the IOP readings to the single individual was: “IOP_Adjusted_ = IOP_Measured_ + 0.054 x (Age (Years)– 64.15) - 0.033 x (Mean Blood Pressure (mmHg) - 90.25) - 0.017 x (Pulse Rate (Beats/min) - 72.56) - 0.426 x(History of Diabetes Mellitus (with 1; without 0) -0.148) + 0.071 x (Refractive Error (Diopters)—(-0.17)) - 0.038 x (Central Corneal Thickness (μm) - 532.3) + 0.756 x (Anterior Corneal Curvature Radius (mm) - 7.61) -0.908”. The mean adjusted IOP was 13.6 ± 2.3 mmHg, and the range of the mean ± 2 standard deviations was 9.0 to 18.1 mmHg (Fig 6).

**Table 5:** Associations (multivariable analysis) of intraocular pressure with systemic parameters and ocular variables in the non-glaucomatous participants of the Beijing Eye Study 2011 (n = 3135), after dropping biochemical blood examination parameters and alcohol consumption quantity.

Parameter	*P*-Value	B	95% CI of B	Beta	VIF
Age (Years)	<0.001	-0.05	-0.06, -0.05	-0.19	1.02
Mean Blood Pressure (mm Hg)	<0.001	0.03	0.03, 0.04	0.17	1.01
Pulse Rate	<0.001	0.02	0.01, 0.03	0.06	1.01
Diabetes or not (based on history plus fast glucose)	0.004	0.43	0.19, 0.67	0.06	1.03
Refractive Error (Diopters)	0.001	-0.07	-0.11, -0.03	-0.05	1.00
Central Corneal Thickness (μm)	<0.001	0.04	0.036, 0.041	0.46	1.02
Anterior Corneal Curvature Radius (mm)	<0.001	-0.76	-1.09, -0.42	-0.07	1.02

95% CI: 95% Confidence Interval; B: Unstandardized Coefficient; Beta: Standardized Correlation Coefficient; VIF: Variance Inflation Factor

## Discussion

In our population-basd study on non-glaucomatous individuals, higher IOP was associated (in multivariate analysis) with younger age, higher blood concentration of glucose and triglycerides, higher mean blood pressure, history of diabetes mellitus, higher pulse rate and higher drinking quantity of alcohol, thicker central corneal thickness, more myopic refractive error, and smaller anterior corneal curvature radius. Mean IOP decreased by 0.50 mmHg mmHg for each decade increase in age. It further decreased by 0.76 mmHg for each increase in corneal curvature radius by 1mm. IOP increased by 0.33 mmHg, 0.17 mmHg, 0.43 mmHg and 0.38 mmHg for each increase in mean blood pressure by 10mmHg, pulse rate by 10 beats/min, presence of diabetes mellitus and central corneal thickness by 10μm, respectively. After adjusting the raw IOP readings for their associations with parameters measurable under routine clinical conditions, the range of IOP within the range of the mean ± 2 standard deviations was between 9.0 and 18.1 mmHg.

The findings confirmed results obtained in previous hospital-based and population-based investigations and extend them for associations of IOP with a panoply of ocular and systemic variables. The mean value of the raw IOP readings of 14.5 ± 2.7 mmHg with a ±2 standard deviations range of 9.2–19.7 mmHg in our study population agreed with the normal range of IOP readings for Caucasians as reported by Leydhecker and colleagues with a mean IOP of 15 to 16 mmHg and a range of 10 to 21 mmHg in their classical study dating back to the year of 1958 [2]. It was also consistent with investigations performed by other researchers [8, 18].

Our study also confirmed previous studies in which higher IOP was correlated with higher blood pressure, such as in the Beaver Dam Study, the Blue Mountains Study, the Tanjong Pagar Study, the Los Angeles Latino Eye Study, the Singapore Malay Eye Study and a recent investigation using the UK biobank [8, 12, 18, 27–29]. The whole array of these studies from different continents shows that the association of IOP with blood pressure is a trans-ethnic finding. It fits with other recent investigations, in which blood pressure, cerebrospinal fluid pressure and IOP were associated with each other [30]. It may suggest a common element in the regulation of all three pressures, as may also be inferred from the results of the experimental investigation carried out by Samuels and colleagues [31]. Samuels and coworkers reported that chemical stimulation of neurons in the dorsomedial and perifornical hypothalamus led to an elevation of IOP and intracranial pressure. The correlation between higher IOP and higher pulse rate fitted with the association between higher IOP and higher blood pressure and further supported the notion of a correlation between blood circulation-related parameters and IOP.

The relationship between higher IOP and thicker central corneal thickness has long been described and several equations have been postulated to correct the measured IOP reading for its dependence on central corneal thickness [16, 32–36]. In the present study, IOP decreased by 0.4 mmHg for each increase in central corneal thickness by 10 μm (Table 3). The value of 0.4 mmHg IOP correction for 10 μm difference in central corneal thickness was exactly the same as found by cannulating the anterior chamber in patients and measuring the IOP directly [34]. Besides central corneal thickness, corneal curvature was another cornea-related factor influencing the IOP readings: The flatter the cornea, the lower were the IOP readings at a given IOP (Tables 3–5) (Fig 5). It was in agreement with the results obtained in the Reykjavik Eye Study and in other investigations [16, 33]. The relationship can be explained geometrically, since a relatively flat cornea as compared to a relatively steep cornea needs less force to be flattened to an area of a defined size. This double relationship between IOP and cornea related parametes may be of importance for corneal refractive surgery, since the latter thins and flattens the cornea. Considering only central corneal thickness and neglecting corneal curvature for adjusting the IOP readings in patients after corneal refractive surgery may therefore underestimate the true IOP.

The association between higher IOP and more myopic refractive error or longer axial length is in agreement with previous studies [16, 18]. The reasons of the associations have remained elusive so far, also since myopic axial elongation affects almost exclusively the posterior half of the globe, while the dimensions of the anterior half of the globe, except for a deepening of the anterior chamber, remain mostly unchanged in axially elongated eyes [37].

The inverse relationship between higher IOP and older age may be of clinical importance [8, 18, 29]. Based on the landmark studies by Leydhecker and others, glaucoma was formerly defined by an IOP higher than 21 mm Hg. Leydhecker´s study was followed by the landmark study by Hollows and colleagues in 1966 by which the concept of the definition of glaucoma as an optic nerve damage was introduced into ophthalmic epidemiology, and by which the notion of glaucoma at normal levels of pressure was promoted [7]. It was confirmed by an increasing number of studies, in particular population-based studies such as the Japanese Tajimi Study, which showed that the majority of patients with glaucomatous optic nerve damage and glaucomatous visual field loss had IOP readings within the normal range of 10 to 21 mm Hg [2, 5]. In the population-based Tajimi Study on 3021 individuals, the prevalence of primary open-angle glaucoma with IOP levels of 21 mmHg or less was considerably higher than the prevalence of primary open-angle glaucoma with IOP readings of more than 21 mmHg (3.6% versus 0.3%) [5]. In 92% patients with primary open-angle glaucoma, the IOP was 21 mmHg or less. The decrease of IOP with older age suggests that the normal range of IPO should be age-adjusted and, if IOP at all is taken as a criterion in the definition of glaucoma, the age-adjusted normal range of IOP may be used for the glaucoma definition. Taking into account the associations of IOP with other parameters, namely blood pressure, pulse rate, refractive error, central corneal thickness and corneal curvature, may further refine the normal range of IOP and may perhaps improve the usefulness of the IOP in the definition of glaucoma. It has however to be taken into account that it widely been acknowledged that the IOP should not form part of the disease definition for glaucoma [38]. The relationship is further complicated as the glaucoma prevalence increases with age.

Limitations of our study should be discussed. First, IOP was measured by air puff non-contact tonometry. Previous studies have shown that IOP readings obtained by non-contact tonometry can differ from those measured by Goldmann applanation tonometry. Second, a major concern in any population-based study is nonparticipation. The participation rate in the Beijing Eye Study 2011 of 78.8% was however relatively high so that it may be unlikely that non-participation had a major effect on the results. Third, our investigation had a cross-sectional design so that the relationships found between the IOP and other parameters could be described only as associations and not as causal relationships. Fourth, several parameters such as socioeconomic factors were assessed in an interview with questionnaires. Fifth, the individuals included into our study and those not included differed in some baseline characteristics such as age, refractive error and axial length (Table 1). These differences were mostly due to the associations between these parameters and glaucoma which was an exclusion criterion of our study.

In conclusion, IOP depends on a multitude of factors including age and blood pressure. These associations may be taken into account in the definition of the normal range of IOP and in the definition of so called normal-pressure glaucoma.
